# *N*-acetyl-seryl-aspartyl-lysyl-proline: a valuable endogenous anti-fibrotic peptide for combating kidney fibrosis in diabetes

**DOI:** 10.3389/fphar.2014.00070

**Published:** 2014-04-14

**Authors:** Keizo Kanasaki, Takako Nagai, Kyoko Nitta, Munehiro Kitada, Daisuke Koya

**Affiliations:** Department of Diabetology and Endocrinology, Kanazawa Medical UniversityUchinada, Ishikawa, Japan

**Keywords:** arb, ACE-I, fibroblast, kidney fibrosis, diabetes mellitus

## Abstract

Fibroproliferative diseases are responsible for 45% of deaths in the developed world. Curing organ fibrosis is essential for fibroproliferative diseases. Diabetic nephropathy is a common fibroproliferative disease of the kidney and is associated with multiorgan dysfunction. However, therapy to combat diabetic nephropathy has not yet been established. In this review, we discuss the novel therapeutic possibilities for kidney fibrosis in diabetes focusing on the endogenous anti-fibrotic peptide, *N*-acetyl-seryl-aspartyl-lysyl-proline (AcSDKP), which is the substrate for angiotensin-converting enzyme and exhibits meaningful anti-fibrotic effects in various experimental models of fibrotic disease.

## INTRODUCTION

Diabetic nephropathy is the leading cause of end-stage renal disease (ESRD) worldwide (Ritz et al., 1999; Viswanathan, 1999; Parving, 2001; Remuzzi et al., 2002). Current treatment strategies can partially slow the progression of the disease, but completely ceasing the progression of diabetic nephropathy is challenging (Lewis et al., 1993; Brenner et al., 2001). Once diabetic nephropathy progresses to ESRD, management with dialysis is associated with increased cardiovascular morbidity and mortality compared to non-diabetic ESRD (Parving, 2001; Remuzzi et al., 2002). Diabetic organ injuries are essentially due to glucose metabolism defects. Therefore, normalizing blood glucose homeostasis is essential for diabetes therapies (The Diabetes Control and Complications Trial Research Group, 1993; Ohkubo et al., 1995; UK Prospective Diabetes Study [UKPDS] Group, 1998). However, recent clinical trials have indicated that the normalization of blood glucose levels is challenging in diabetes owing to an increased mortality risk, which is likely associated with frequent hypoglycemia (Ismail-Beigi et al., 2010). Consistent with this problem, blood glucose-lowering strategies aimed at normalizing the blood glucose level resulted in an increased mortality for the patients recruited to the intensive therapy group of the ACCORD trial (Ismail-Beigi et al., 2010). Therefore, to ameliorate the mortality associated with diabetic complications, additional therapeutic strategies to those that target proper blood glucose control are required.

Fibrosis is the final common pathway of progressive kidney diseases (similar to what occurs in other organs) and results in the destruction of the normal kidney structure and a significant deterioration in kidney function (Risdon et al., 1968; Schainuck et al., 1970; Striker et al., 1970; Mackensen-Haen et al., 1981; Nath, 1992; Kanasaki et al., 2012). Kidney fibrosis is induced by prolonged damage associated with impairment of the normal regulatory mechanisms for wound healing and an accumulation of extracellular matrix (ECM). Kidney fibroblasts play an important role in this fibrotic process, but the origin of the fibroblasts remains unclear and has become the focus of intense debate (He et al., 2013; Kanasaki et al., 2013). Despite such a controversial discussion, significant heterogeneity for the matrix-producing fibroblasts is thought to exist (Kanasaki et al., 2013), and diverse origins for the fibroblasts have been described, such as resident fibroblasts, resident pericytes, epithelial-to-mesenchymal transition (EMT), and endothelial-to-mesenchymal transition (EndMT) (Kanasaki et al., 2013). The activation of such fibroblasts is important for the development of matrix-producing fibroblasts, and inhibiting this process could be a promising therapeutic target for diabetic kidney disease.

*N*-acetyl-seryl-aspartyl-lysyl-proline (AcSDKP), an endogenous anti-fibrotic peptide, is a substrate for angiotensin-converting enzyme, and the plasma level of AcSDKP has been shown to increase by fivefold after acute administration of the ACE-inhibitor (ACE-I) captopril (Azizi et al., 1996). In this review, we focus on the use of AcSDKP to treat diabetic kidney disease by analyzing the potential mechanisms involving AcSDKP.

## AcSDKP SYNTHESIS

AcSDKP is a tetrapeptide originally isolated from fetal calf bone marrow (Lenfant et al., 1989), and studies have recently focused on its anti-fibrotic property.

**FIGURE 1:**
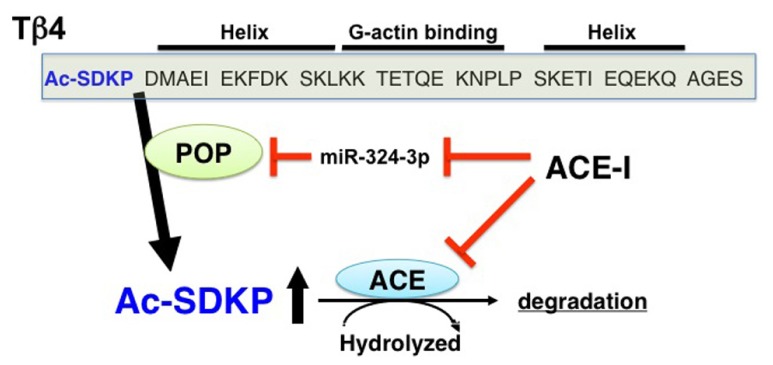
**Synthesis and metabolism of AcSDKP.** Tβ4, a G-actin binding peptide, is cleaved by POP, and subsequently its N-terminal tetrapeptide, AcSDKP, is synthesized. AcSDKP is hydrolyzed and degraded by ACE. ACE-I may suppress miR-324-3p, which may inhibit protein expression of POP. Therefore, the mechanisms underlying the increased levels of AcSDKP by ACE-I may include both the suppression of degradation pathway and the induction of synthesis pathway of AcSDKP.

The details for the synthetic pathways responsible for the endogenous synthesis of AcSDKP are not yet clear, but the available information strongly suggests that thymosin β4 (Tβ4), one of the G-actin-sequestering peptides, is the most likely candidate for the AcSDKP precursor (Grillon et al., 1990; Liu et al., 2010; **Figure 1**). In HeLa cells, when Tβ4 was knocked down using Tβ4 small interfering (si)RNA, there was significant suppression of AcSDKP expression (Liu et al., 2010). Furthermore, Lenfant and colleagues elegantly demonstrated that incubating radiolabeled [^3^H] Tβ4 with bone marrow cells or bone marrow lysate results in the formation of [^3^H]AcSDKP (Grillon et al., 1990). AcSDKP is the N-terminal sequence of Tβ4 (**Figure 1**), and AcSDKP was thought to be synthesized by cleavage employing Asp-N endopeptidase (Grillon et al., 1990). However, Asp-N is found only in bacteria and not in vertebrates. Therefore, Cavasin et al. (2004) investigated other enzymes that may be responsible for Tβ4-mediated AcSDKP production, and they identified that prolyl oligopeptidase (POP; in some papers described as prolyl endopeptidase, PREP) is responsible for Tβ4-mediated AcSDKP production (Cavasin et al., 2004; **Figure 1**).

In addition to AcSDKP, the Tβ4 precursor peptide displays anti-fibrotic and tissue-protective effects. Tβ4 is a 43 amino acid peptide (4.9 kDa) that can sequester G-actin and regulate its polymerization to F-actin (Huff et al., 2001; Hannappel, 2010). Tβ4 is expressed ubiquitously and exhibits various biologically significant activities (Huff et al., 2001; Hannappel, 2010). The utility of Tβ4 has been shown by Bock-Marquette et al. (2004), whereby exogenous intracardiac and intraperitoneal Tβ4 administration significantly restored cardiac function by neovascularization in an experimental myocardial infarction mouse model. Cardiac function restoration by Tβ4 has also been shown to occur by epicardial progenitor mobilization (Smart et al., 2007). Together, these reports suggest that Tβ4 exhibits organ-protective effects associated with anti-fibrosis and enhanced angiogenesis. It is unknown how AcSDKP contributes to Tβ4-mediated organ protection, but a recent paper has suggested that the anti-fibrotic effects of Tβ4 were lost when POP was inhibited in unilateral ureteral obstruction (UUO) models (Zuo et al., 2013).

## METABOLISM OF AcSDKP AND ACE STRUCTURE

**FIGURE 2:**
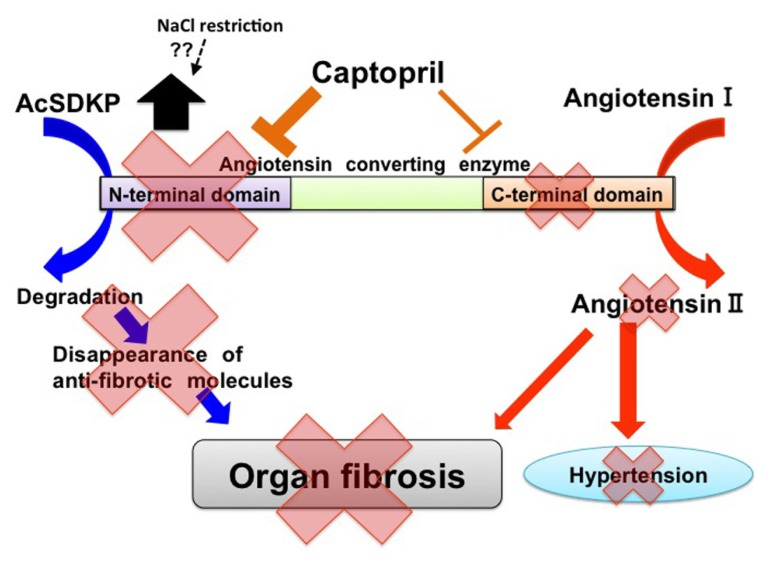
**Two catalytic domains of ACE and biological roles of ACE in tissue fibrosis.** In ACE, there are two catalytic sites. Angiotensin I exerts higher affinity for the C-terminal catalytic site of ACE. AcSDKP is a substrate for the N-terminal domain. ACE may induce tissue fibrosis by both the production of angiotensin II and the decreased level of AcSDKP. Captopril exhibits higher affinity for the N-terminal catalytic sites of ACE when compared with C-terminal catalytic sites. Furthermore, NaCl restriction on top of RAS-blockade may increase AcSDKP levels by unknown mechanisms.

As mentioned above, AcSDKP is produced by POP from the N-terminal peptide sequence of Tβ4, and AcSDKP is hydrolyzed in the presence of ACE (**Figure 1**). The plasma level of AcSDKP is minimal in normal conditions, and the AcSDKP concentration increased fivefold following administration of captopril (Azizi et al., 1996). For ACE, there are two catalytic domains, namely the N-terminus and C-terminus, which contain the HEMGH consensus amino acid sequence. This motif is responsible for binding zinc and is crucial for enzymatic activity. These catalytic domains are responsible for the cleavage of target substrates (**Figure 2**; Wei et al., 1991; Bernstein et al., 2011). The overall amino acid homology between these two ACE catalytic domains is approximately 60%, and the homology reaches approximately 89% in the portions involved in catalysis (Bernstein et al., 2011). Studies of the genomic DNA exons and exon–intron boundaries have suggested that the ACE gene in higher organisms is the result of an ancient gene duplication event (Hubert et al., 1991). The resultant ACE, which has two catalytic sites, is a so-called somatic ACE, an isozyme present in the plasma and generated by the endothelium, kidneys, and other somatic tissues. By contrast, the testis ACE, an ACE composed of only the C-terminal domain and not the N-terminal domain, is only expressed by developing male germ cells and is a smaller protein. This testis ACE, which lacks the N-terminal domain, is important because male mice lacking testis ACE exhibit fertility problems compared to wild-type mice (Krege et al., 1995; Esther et al., 1997; Fuchs et al., 2005). The testis ACE has been suggested to be the primordial form of ACE (Bernstein et al., 2011). These two ACE isozymes result from two separate promoter regions in the ACE gene (Howard et al., 1990; Langford et al., 1991).

Such ACE gene duplication is suspected to have occurred early in evolution and might have occurred before mammalian differentiation (Hubert et al., 1991). These two catalytic domains have been suggested to be functionally different (**Figure 2**). An ACE or an ACE-like enzyme is present in mammals, fish, worms, insects, crabs, and even ticks (Coates et al., 2000; Macours and Hens, 2004). Two ACE homologs, Ance and Acer, in *D. melanogaster* (Cornell et al., 1995; Houard et al., 1998; Bingham et al., 2006; Akif et al., 2010) have been studied comprehensively. In *D. melanogaster*, each of these enzymes has a single catalytic domain. An analysis of the gene structure and the enzymatic properties of each protein has revealed that Ance is similar to the C-terminal domain of somatic ACE and that Acer is more similar to the N-terminal domain of somatic ACE. Studies have suggested that the ACE gene duplication in vertebrates occurred approximately 330–350 million years ago (Cornell et al., 1995). Both the N-terminal and C-terminal catalytic domains of ACE have been conserved over a longer period of time, thus suggesting that each ACE domain displays an important and different physiologic role.

Bradykinin is hydrolyzed at approximately the same ratio via both of these catalytic domains. Additionally, either the N-terminal or C-terminal catalytic domain can cleave angiotensin-I. The C-terminal domain has a fivefold higher affinity for angiotensin I (**Figure 2**; Wei et al., 1991; Rousseau et al., 1995; Bernstein et al., 2011). The affinity of angiotensin-I for the ACE catalytic site results in an interesting profile for blood pressure homeostasis. Mutant mice for each catalytic site of ACE, including the N-terminal (ACE^N^-KO; Fuchs et al., 2004) and C-terminal (ACE^C^-KO; Fuchs et al., 2008) domains, have normal blood pressure, but the underlying mechanisms by which blood pressure can be maintained are different, which is mainly due to the affinity of angiotensin I for the ACE catalytic domain. ACE^N^-KO mice have similar plasma levels of angiotensin I and renin compared to wild-type mice (Fuchs et al., 2004). In ACE^N^-KO mice, the C-terminal catalytic ACE domain is intact. Normal levels of angiotensin I and renin with a normal blood pressure indicate that the C-terminal domain of ACE is responsible for the majority of angiotensin II production in a normal state, thus resulting in no compensatory induction of renin or angiotensin I to maintain blood pressure in the ACE^N^-KO mice (Fuchs et al., 2004). In ACE^C^-KO mice, the mice with an intact N-terminal catalytic ACE domain have normal blood pressure similar to ACE^N^-KO mice, but the underlying mechanisms are completely different (Fuchs et al., 2008). Interestingly, the ACE^C^-KO mice have a significantly higher plasma angiotensin I level (approximately sevenfold) and plasma renin concentration (2.6-fold) compared to wild-type mice suggesting relatively lower catalytic effects of the N-terminal domain on angiotensin II production. Such an insufficient catalytic ability for converting angiotensin I to angiotensin II by the N-terminal ACE domain results in the compensatory induction of renin and subsequent elevation of angiotensin I. As a result, the angiotensin II levels are maintained to keep the blood pressure at normal levels.

**FIGURE 3:**
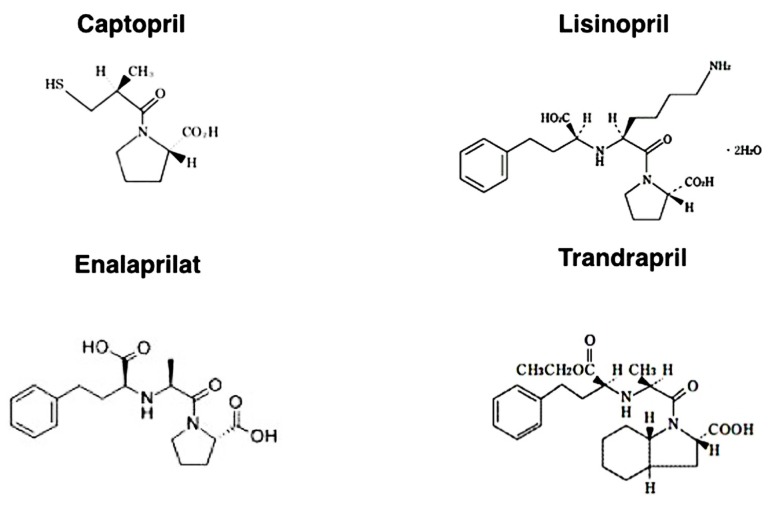
**Chemical structure of ACE inhibitors**.

In this regard, the affinity of each ACE-I for the ACE catalytic domains is structure dependent (**Figure 3**). The hydrophobic moieties of ACE-Is have been suggested to play an essential role in domain selectivity (Zisman, 1998). For instance, captopril, the first ACE inhibitor used clinically, exhibits approximately a threefold greater affinity for the N-terminal domain compared to the C-terminal domain. By contrast, the relatively newer ACE-Is, such as enalaprilat, lisinopril, and trandolapril, display a higher affinity (approximately 4–20 times higher) for the C-terminal domain (Acharya et al., 2003) because these drugs were developed as anti-hypertensive drugs (chemical structures of each ACE-I are shown in **Figure 3**).

AcSDKP is hydrolyzed only by the N-terminal catalytic ACE domain (**Figure 2**). In fact, the testis (where the germinal-type ACE is expressed) is associated with a higher level of AcSDKP relative to other tissues (Stephan et al., 2000; Fuchs et al., 2004). The role of the N-terminal ACE domain in the degradation of AcSDKP and its biological significance have been successfully reported by Li et al. (2010), who analyzed bleomycin-induced lung injury in wild-type, ACE^C^-KO, and ACE^N^-KO mice. The ACE^N^-KO mice had significantly less bleomycin-induced lung fibrosis as analyzed by lung histology and hydroxyproline level. Such protection against bleomycin-induced injury was not found in the ACE^C^-KO mice. Because the ACE^N^-KO mice had an elevated level of AcSDKP, the authors examined the effects of S-17092, a POP inhibitor. As mentioned above, POP is the enzyme responsible for AcSDKP production from Tβ4. As expected, the S-17092-treated ACE^N^-KO mice developed lung fibrosis similar to the wild-type mice. AcSDKP administration to the wild-type mice reduced bleomycin-induced lung fibrosis. This study revealed that AcSDKP elevation caused by inhibition of the N-terminal catalytic ACE domain leads to significant endogenous anti-fibrosis signaling in the lungs (Li et al., 2010). Therefore, an N-terminal catalytic domain-specific ACE-I, such as RXP407 (**Figure 2**), may have great potential as an antifibrotic therapy (Junot et al., 2001; Vazeux et al., 2001; Kroger et al., 2009; Anthony et al., 2010).

## CELL CYCLE CONTROL AND AcSDKP

AcSDKP is a naturally occurring inhibitor of hematopoietic stem cell proliferation that prevents entry into the S phase from G1 in the cell cycle (Wdzieczak-Bakala et al., 1990). The effect of AcSDKP on cell proliferation is not limited to hematopoietic stem cells, and AcSDKP has been shown to inhibit human mesangial cell proliferation (Kanasaki et al., 2006) as well as renal (Iwamoto et al., 2000) and cardiac fibroblast (Rhaleb et al., 2001a) proliferation. Moreover, AcSDKP has been shown to inhibit collagen deposition in mouse cardiac fibroblasts (Rhaleb et al., 2001a). The detailed mechanisms for AcSDKP-mediated cell cycle regulation are not yet clear, but AcSDKP may inhibit serum-stimulated extracellular signal-regulated kinase (ERK) signaling. Alternatively, as we have shown in mesangial cells, AcSDKP may inhibit cell cycle progression/DNA synthesis stimulated by serum or platelet-derived growth factor-B in human mesangial cells through the induction of cell cycle regulators, such as p53, p27^kip1^, and p21^cip1^, together with the inhibition of cyclin D1 (Kanasaki et al., 2006). Additionally, p53 induces the expression of p21^cip1^ and p27^kip1^, together with inhibition of the cell cycle at G1/S (Kanasaki et al., 2006). In mesangial cells, p53 is a key regulator for the induction of these cell cycle modulators (Kanasaki et al., 2006). Supporting the role of p53 in cell cycle inhibition, AcSDKP inhibits cell cycle progression in normal cells, but AcSDKP does not suppress progression in chronic myeloid leukemia (CML) progenitors in long-term culture (Cashman et al., 1994) as these cells frequently exhibit p53 deficiency (Chen et al., 1990; Feinstein et al., 1991; Bi et al., 1992). Indeed, siRNA-mediated gene silencing of p53 in human mesangial cells has been shown to abolish AcSDKP-mediated cell cycle inhibition of mesangial cells proliferation (Kanasaki et al., 2006). These data suggest that AcSDKP inhibits mesangial cell proliferation through p53 induction.

## ANTI-FIBROTIC EFFECTS OF AcSDKP

AcSDKP reportedly exhibits anti-fibrotic organ-protective effects in various experimental models (Fromes et al., 2006; Omata et al., 2006; Castoldi et al., 2013; Zuo et al., 2013). Additionally, we have reported that AcSDKP prevents mesangial matrix expansion in diabetic db/db mice (Shibuya et al., 2005). Cavasin et al. (2007) reported that endogenous levels of AcSDKP play an important role for anti-fibrotic effects. Fibroblasts play an important role in tissue fibrosis. As mentioned previously, AcSDKP has been shown to suppress the proliferation of renal (Iwamoto et al., 2000) and cardiac fibroblasts (Rhaleb et al., 2001a).

The local accumulation of pro-fibrotic cytokines in the microenvironment following kidney insult results in ECM-producing cell activation, which is essential for renal fibrogenesis. The fundamental matrix-producing cells, which generate a large quantity of interstitial matrix components (including fibronectin and type I and type III collagens), are indeed fibroblasts (Strutz and Zeisberg, 2006). Activated fibroblasts (or myofibroblasts) would be an important source of ECM-producing renal cells, but almost all cell types (either resident or non-resident kidney cells) are responsible for ECM production (Kanasaki et al., 2013). Those cells include resident fibroblasts, tubular epithelial cells, vascular smooth muscle cells, and a subset of invading macrophages. In such a process, the profibrotic cytokine, transforming-growth factor-(TGF)-β, has a fundamental role. Consistent with this information, blocking either TGF-β or the TGF-β-stimulated Smad transcriptional factor signaling pathway has been shown to exhibit anti-fibrotic effects (Border and Noble, 1994; Miyazono, 2000; Kanasaki et al., 2003, 2011; RamachandraRao et al., 2009; Takakuta et al., 2010; Hills and Squires, 2011; Lan, 2011; Sharma et al., 2011; Choi et al., 2012). In fibrotic kidneys, activated fibroblasts express α smooth muscle actin (αSMA) and are often called myofibroblasts, which display unique contractile properties (Strutz and Zeisberg, 2006). The renal myofibroblast is thought to be an activated fibroblast that plays a role during kidney fibrosis. Thus, numerous studies have been performed to analyze the origin, activation, and regulation of these matrix-producing myofibroblasts (Grande and Lopez-Novoa, 2009; Meran and Steadman, 2011).

**FIGURE 4:**
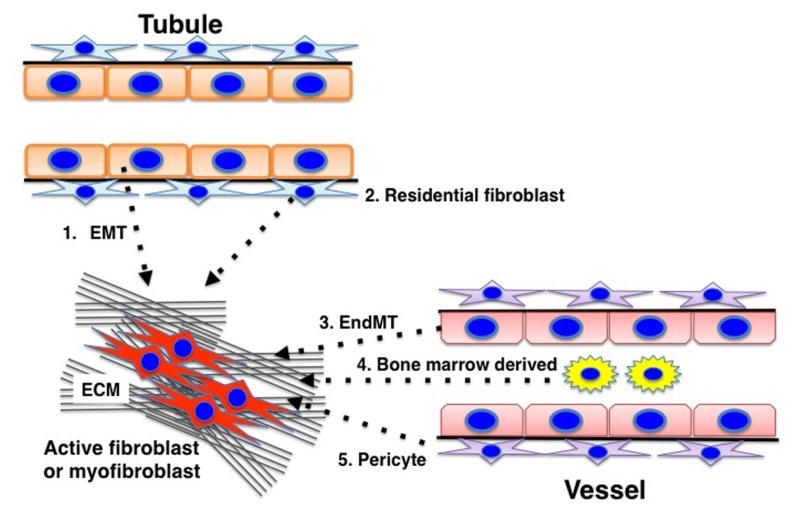
**Diverse origins of myofibroblasts.** Kidney fibrosis is a well-coordinated event originating from various sources: **(1)** tubular epithelial cells; **(2)** interstitial resident fibroblasts; **(3)** endothelial cells; **(4)** bone marrow-derived cells; and **(5)** pericytes that contribute to myofibroblast activation/formation.

There are five well-reported sources of matrix-producing myofibroblasts (**Figure 4**), including activated resident fibroblasts, differentiated pericytes, recruited circulating bone marrow-derived cells, and mesenchymal cells transformed from tubular epithelial cells or endothelial cells (Barnes and Gorin, 2011). There are intense debates regarding such diverse myofibroblast-generating pathways and their contribution in renal fibrosis (Zeisberg and Duffield, 2010). However, even though many studies focused on analyzing the number of myofibroblast and their origin, the most important clue to understand kidney fibrosis is the functional interaction and effects of these fibroblasts and resident kidney cells. Thus, Kalluri and colleagues reported a breakthrough observation regarding the origin and function of kidney myofibroblasts (LeBleu et al., 2013). In a recent publication analyzing a UUO model by LeBleu et al. (2013), the accumulation of myofibroblasts in the kidneys arose from predominantly two different origins as follows: local proliferation of resident kidney fibroblasts (~50%) and bone marrow-derived cells without any evidence of proliferation in the kidney (35%). Bone marrow-derived mesenchymal stem cells can differentiate into myofibroblasts in the presence of TGF-β1. Surprisingly, while the loss of *Tgfbr2* in αSMA^+^ cells led to an approximately 56% reduction in the accumulation of myofibroblasts, only an approximately 29% reduction in kidney fibrosis was found. Additionally, F4/80^+^ and CD11b^+^ macrophage recruitment was significantly reduced in mice lacking *Tgfbr2* in their αSMA^+^ cells. The loss of *Tgfbr2 in* αSMA^+^ cells likely affects myofibroblasts specifically derived through differentiation.

The administration of AcSDKP ameliorated kidney fibrosis and glomerular sclerosis in hypertensive rats as well as in diabetic and non-diabetic kidney disease models without altering blood pressure (Peng et al., 2001; Rhaleb et al., 2001b). While many reports have consistently shown strong anti-fibrotic effects *in vivo* and the direct effects of AcSDKP on culture fibroblast *in vitro*, it is still unclear how AcSDKP affects fibroblast activation or differentiation into myofibroblasts. There were two publications that describe the association between AcSDKP and myofibroblast differentiation. The first report by Peng et al. (2010) found that human cardiac fibroblasts treated with TGF-β1 transform into myofibroblasts as indicated by increased expression of αSMA and a higher expression of the embryonic isoform of smooth muscle myosin compared to untreated cells, and this report also demonstrated that AcSDKP inhibited TGF-β1-induced differentiation of cardiac fibroblasts into myofibroblasts. The second report by Xu et al. (2012) demonstrated that AcSDKP inhibits the TGF-β1-induced pulmonary fibroblast transformation into myofibroblasts and myofibroblast localization in siliconic nodules in the lung. These reports described the suppressive effects of AcSDKP on myofibroblast differentiation, but further investigations are needed to reveal both the specific origin of myofibroblasts and specific target molecules affected by AcSDKP. Regard with this, we have recently shown that AcSDKP may inhibit EndMT via restoration of fibroblast growth factor receptor (FGFR) and FGFR-associated induction of microRNA let-7, the critical factors for the maintenance of endothelial homeostasis (Chen et al., 2012), in diabetic mice kidneys (Nagai et al., 2014).

**FIGURE 5:**
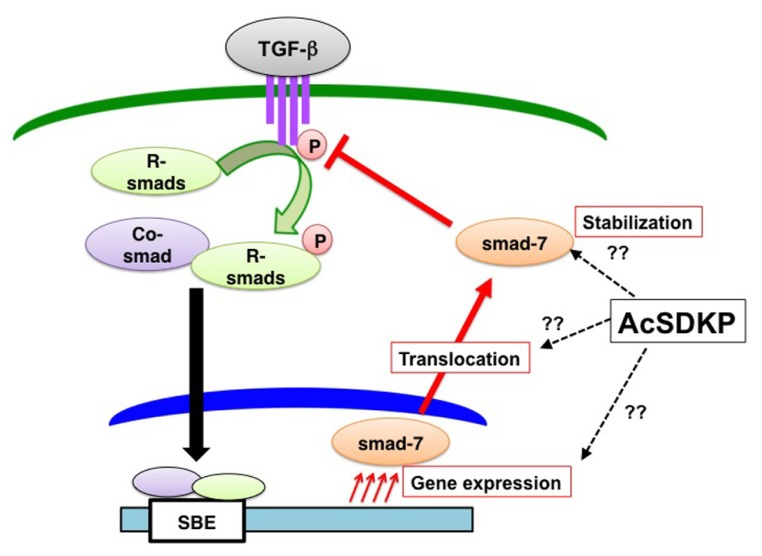
**AcSDKP is an anti-TGF-β/Smad peptide.** Once TGF-β binds to TGF-β receptors on the cell membrane, the TGF-β and TGF-β-receptor interaction induces phosphorylation of receptor-regulated (R)-Smads. Phosphorylated R-Smads interact with the common (co)-Smad in the cytoplasm. These Smad heterodimers in the nucleus then bind to the genomic promoter region of DNA, which is called the Smad-binding element (SBE). AcSDKP may induce Smad7 gene expression, protein stabilization, or translocation from the nucleus of cells to the cytoplasm as well as inhibit phosphorylation of R-Smads by TGF-β receptors. However, detailed mechanisms are not yet known.

We and others have shown that AcSDKP inhibits TGF-β-induced Smad2 phosphorylation (**Figure 5**), and the anti-TGF-β/Smad pathway is the key to understand its antifibrotic effect (Pokharel et al., 2002; Kanasaki et al., 2003). Additionally, this observation identifies AcSDKP as the first endogenous circulatory molecule that specifically inhibits TGF-β-induced receptor regulated (R)-Smad phosphorylation. The Smads are transcription factors specific to the TGF-β superfamily and play a central role in signal transduction from cell membrane receptors (Border and Noble, 1994; Miyazono, 2000). Depending on their role in signaling, Smads are classified into three categories as follows: (a) receptor-regulated Smads, or R-Smads (Smad2 and 3); (b) common Smad, or co-Smad (Smad4); and (c) inhibitory Smads, or I-Smads (Smad6 and 7). Once TGF-β binding occurs, the type II receptor physically interacts with the type I receptor inducing the phosphorylation of serine residues on the type I receptor (**Figure 5**; Wrana et al., 1992). The phosphorylated type I receptor phosphorylates R-Smads, and phosphorylated R-Smads subsequently interact with co-Smads in the cytoplasm (**Figure 5**). The R-Smad and co-Smad heterodimers then translocate into the nucleus (**Figure 5**) with the help of importin-β (Xiao et al., 2000; Kurisaki et al., 2001). The Smad heterodimers bind to the Smad-binding elements of the target promoter DNA regions (**Figure 5**). By contrast, I-Smads localize to the nucleus (**Figure 5**; Kanasaki et al., 2003), and nuclear-localized I-Smads translocate to the cytoplasm following TGF-β stimulation. I-Smad is believed to competitively inhibit R-Smad phosphorylation by the type I receptor or induce ubiquitination of the receptors by I-Smad interaction with E3 ligase smurf proteins (Nakao et al., 1997; Ebisawa et al., 2001; Gronroos et al., 2002; Suzuki et al., 2002).

Although the underlying mechanisms by which AcSDKP inhibits TGF-β-induced phosphorylation of R-Smad are not completely known, I-Smads are likely involved at least in part (**Figure 5**). We have shown that the incubation of human mesangial cells in the presence of AcSDKP results in the cytoplasmic translocation of Smad7 (one of the I-Smads) in the absence of TGF-β stimulation (**Figure 5**; Kanasaki et al., 2003). Several studies have reported an increased Smad7 level *in vivo* following AcSDKP administration supporting the Smad7-mediated anti-TGF-β/Smad effects of AcSDKP (**Figure 4**; Omata et al., 2006; Lin et al., 2008). Alternatively the suppression of TGF-β type I receptor levels via microRNA let-7 (Chen et al., 2012) induced by AcSDKP (Nagai et al., 2014) may contribute to the AcSDKP-inhibited R-Smad phosphorylation.

## AcSDKP AND APOPTOSIS

AcSDKP was originally identified as a regulator of hematopoietic stem cells (Lenfant et al., 1989; Pradelles et al., 1990, 1991). AcSDKP can suppress apoptosis of hematopoietic cells induced by cytotoxic stresses, such as chemotherapy (Bogden et al., 1991; Grillon et al., 1993), radiation (Watanabe et al., 1996; Deeg et al., 1997), high temperature (Wierenga and Konings, 1994; Wierenga et al., 1998, 2000), and photofrin II-mediated phototherapy (Coutton et al., 1994). Enhanced apoptosis is linked to tissue fibrosis, and inhibition of the apoptosis pathway has been associated with anti-fibrosis therapy in several organs (Gieling et al., 2008; Coward et al., 2010; Rodriguez-Iturbe and Garcia Garcia, 2010; Dooley et al., 2011).

## AcSDKP AND INFLAMMATION

Inflammation is essential for tissue repair, except in embryos where tissue repair can be completed without typical inflammation (Bullard et al., 2003; Redd et al., 2004). In adults, organ inflammation is closely linked to tissue repair, the regeneration of parenchymal cells and filling in tissue defects with fibrous tissue, such as scar formation (Wynn, 2007). Moreover, progressive fibrosis with sustained inflammation is recognized as a type of chronic wound with normal wound healing defects (Liu, 2011). In experimental animal models, the amelioration of tissue fibrosis by AcSDKP has been associated with inhibition of inflammation in the kidneys, heart, and liver (Yang et al., 2004; Omata et al., 2006; Peng et al., 2007; Lin et al., 2008; Sharma et al., 2008; Liu et al., 2009b; Chen et al., 2010). AcSDKP suppresses monocyte chemoattractant protein-1 (MCP-1; Wang et al., 2010), one of the key chemokines that regulates macrophage infiltration. AcSDKP has been shown to inhibit the key pro-inflammatory transcriptional factor, NFκB, and associated chemokines (Nakagawa et al., 2012; Gonzalez et al., 2014). However, another report has described AcSDKP-induced MCP-1 expression and an accumulation of Mac1-positive cells in a model of surgically induced hind-limb ischemia (Waeckel et al., 2006). In their report, AcSDKP-induced MCP-1 expression is the key for AcSDKP-mediated tissue repair and post-ischemic neovascularization based on MCP-1 knockout mice (Waeckel et al., 2006), thereby suggesting that AcSDKP does not simply inhibit inflammation but may regulate normal tissue repair and appropriately control inflammation.

## AcSDKP AND ANGIOGENESIS

Angiogenesis is essential for tissue homeostasis and to promote tissue repair. AcSDKP (Liu et al., 2003; Wang et al., 2004; Fromes et al., 2006) and its precursor peptide, Tβ4 (Malinda et al., 1997; Huff et al., 2001; Koutrafouri et al., 2001; Philp et al., 2003) have been shown to enhance angiogenesis and exhibit anti-fibrotic effects associated with normalization of organ function (Smart et al., 2007). AcSDKP improves skin flap survival and accelerates wound healing (Fromes et al., 2006). The association between tumor angiogenesis and the levels of Tβ4 and AcSDKP has been studied by Wdzieczak-Bakala et al. (1990), and these authors have proposed that high levels of Tβ4 and AcSDKP are linked to tumor progression in hematologic malignancies (Liu et al., 2006, 2008, 2009a, 2010). Angiogenesis plays a pivotal role in cancer development (Nyberg et al., 2005; Folkman, 2007), and the AcSDKP level has been shown to be higher in hematologic malignancies and solid neoplasms (Liu et al., 2006, 2008, 2009a, 2010). An association between the AcSDKP level and tumor angiogenesis was observed in these previous studies, but the pathophysiological significance of this result was not clearly shown.

## PERSPECTIVE

As described above, AcSDKP has emerged as an attractive anti-fibrotic molecule to combat fibroproliferative diseases, including diabetic nephropathy. However, other than its production from Tβ4 by POP and degradation by ACE, the physiological regulation of AcSDKP and its significance in pathogenesis are largely unknown. In this regard, recent publications have provided new clues about the regulation of AcSDKP in experimental animals and in patients treated with ACE-I.

Recently, microRNAs have been implicated as key players in physiological homeostasis, and dysregulation of microRNAs results in pathological conditions, such as tissue fibrosis (He et al., 2013; Srivastava et al., 2013). A fibroblast-activating pathway has also been shown to be associated with microRNA dysregulation (He et al., 2013; Srivastava et al., 2013). Macconi et al. (2012) recently found that one of the microRNAs, miR-324-3p, is significantly increased in the glomeruli of Munich Wistar Frömter (MWF) rats, which is a model for spontaneous progressive nephropathy, and they reported that increased expression of miR-324-3p is present in glomerular podocytes, parietal cells in Bowman’s capsule, and most abundantly in cortical tubules. Interestingly, the predicted target for miR-324-3p is POP, and overexpression of a miR-324-3p mimetic in culture decreased POP protein expression (**Figure 1**). High miR-324-3p expression in MWF rats was associated with reduced POP expression in glomeruli and tubules as well as suppressed urine AcSDKP levels and increased collagen deposition. Surprisingly, the ACE-I lisinopril, suppressed miR-324-3p expression and subsequently increased renal POP expression as well as plasma and urine AcSDKP levels, which were associated with the restoration of a normal kidney structure. This report revealed that the endogenous AcSDKP synthesis pathway is indeed enhanced by ACE-I, regulated by miR-324-3p suppression and associated with induction of POP, the key enzyme for AcSDKP synthesis.

Another important finding in AcSDKP regulation has been reported in a recent clinical trial. Sodium intake has been shown to worsen the clinical outcome of renal diseases (Vegter et al., 2012). Kwakernaak et al. (2013) focused on potential organ-protective effects of AcSDKP and investigated whether sodium restriction in addition to renin–angiotensin system (RAS) blockade results in increased levels of AcSDKP. These authors enrolled 46 non-diabetic chronic kidney disease patients (age 50 ± 13 years) with overt proteinuria and mild to moderate renal insufficiency. The patients were analyzed using a crossover design and subjected to a double-blind experiment for a 6-week study period with a regular sodium diet (194 ± 49 mmol sodium/day) or a low sodium diet (102 ± 52 mmol sodium/day) and either lisinopril (40 mg/day; single RAS-blockade) or lisinopril plus valsartan (320 mg/day; dual blockade). Surprisingly, they found that sodium restriction significantly increased the plasma level of AcSDKP during either single or dual RAS-blockade (**Figure 2**). The AcSDKP level was associated with sodium restriction but independent of sex, age, renal function, blood pressure, body mass index, single RAS-blockade, dual RAS-blockade, treatment sequence, or other dietary factors (calcium and protein intake). This report is indeed surprising because sodium restriction would decrease the circulatory plasma volume, and a decreased plasma volume may be associated with an enhanced RAS feedback, thus resulting in a suppressed AcSDKP level. To understand how sodium restriction in addition to RAS-blockade may alter the AcSDKP level, further investigation is needed. Nevertheless, Kwakernaak et al.’s (2013) study showed the novel regulation of AcSDKP by a mechanism other than an ACE-I in humans. The association between salt intake and AcSDKP levels without RAS-blockade remains unknown and requires future analysis (Kwakernaak et al., 2013). This study may provide some hints for the physiological regulation of AcSDKP in humans, and such knowledge may reveal the AcSDKP level required for anti-fibrotic effects in human kidney diseases, including diabetic nephropathy.

## CONCLUSION

**FIGURE 6:**
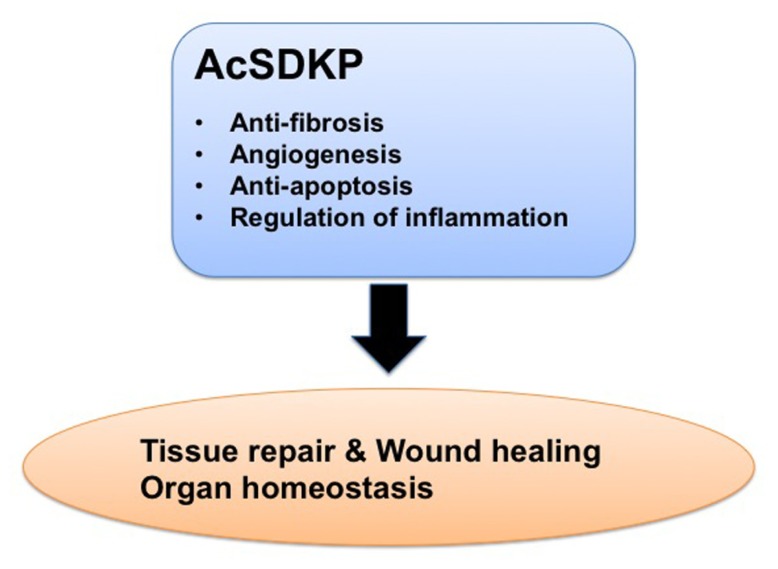
**Beneficial effects of AcSDKP in the process of tissue repair.** AcSDKP exhibits multiple functions shown above, such as regulation of inflammation as well as anti-fibrotic, anti-apoptotic, and pro-angiogenic activities. Therefore, AcSDKP could be a candidate target molecule to combat kidney fibrosis in diabetes.

In this review, we summarized the findings regarding AcSDKP focusing on its physiological regulation, function, and potential as an anti-fibrotic drug. The beneficial effects of AcSDKP could be significant for treating patients with fibroproliferative diseases, including diabetic nephropathy. Clearly, future studies will be required to establish how we can utilize the attractive anti-fibrotic effects of AcSDKP in the clinic and to monitor safety profiling of AcSDKP use. Nevertheless, AcSDKP will emerge as a valuable anti-fibrotic endogenous molecule (**Figure 6**) with the potential to cure devastating fibroproliferative diseases, including diabetic nephropathy.

## Conflict of Interest Statement

The authors declare that the research was conducted in the absence of any commercial or financial relationships that could be construed as a potential conflict of interest.

## References

[B1] Acharya K. R. Sturrock E. D. Riordan J. F. Ehlers M. R. (2003). Ace revisited: a new target for structure-based drug design. *Nat. Rev. Drug Discov.* 2 891–902 10.1038/nrd1227 14668810 PMC7097707

[B2] Akif M. Ntai I. Sturrock E. D. Isaac R. E. Bachmann B. O. Acharya K. R. (2010). Crystal structure of a phosphonotripeptide K-26 in complex with angiotensin converting enzyme homologue (AnCE) from *Drosophila melanogaster*. *Biochem. Biophys. Res. Commun.* 398 532–536 10.1016/j.bbrc.2010.06.113 20599761

[B3] Anthony C. S. Corradi H. R. Schwager S. L. Redelinghuys P. Georgiadis D. Dive V. (2010). The N domain of human angiotensin-I-converting enzyme: the role of *N*-glycosylation and the crystal structure in complex with an N domain-specific phosphinic inhibitor, RXP407. *J. Biol. Chem.* 285 35685–35693 10.1074/jbc.M110.167866 20826823 PMC2975193

[B4] Azizi M. Rousseau A. Ezan E. Guyene T. T. Michelet S. Grognet J. M. (1996). Acute angiotensin-converting enzyme inhibition increases the plasma level of the natural stem cell regulator *N*-acetyl-seryl-aspartyl-lysyl-proline. *J. Clin. Invest.* 97 839–844 10.1172/JCI118484 8609242 PMC507123

[B5] Barnes J. L. Gorin Y. (2011). Myofibroblast differentiation during fibrosis: role of NAD(P)H oxidases. *Kidney Int.* 79 944–956 10.1038/ki.2010.516 21307839 PMC3675765

[B6] Bernstein K. E. Shen X. Z. Gonzalez-Villalobos R. A. Billet S. Okwan-Duodu D. Ong F. S. (2011). Different in vivo functions of the two catalytic domains of angiotensin-converting enzyme (ACE). *Curr. Opin. Pharmacol.* 11 105–111 10.1016/j.coph.2010.11.001 21130035 PMC3075415

[B7] Bi S. Hughes T. Bungey J. Chase A. De Fabritiis P. Goldman J. M. (1992). p53 in chronic myeloid leukemia cell lines. *Leukemia* 6 839–842 1640738

[B8] Bingham R. J. Dive V. Phillips S. E. Shirras A. D. Isaac R. E. (2006). Structural diversity of angiotensin-converting enzyme. *FEBS J.* 273 362–373 10.1111/j.1742-4658.2005.05069.x 16403023

[B9] Bock-Marquette I. Saxena A. White M. D. Dimaio J. M. Srivastava D. (2004). Thymosin beta4 activates integrin-linked kinase and promotes cardiac cell migration, survival and cardiac repair. *Nature* 432 466–472 10.1038/nature03000 15565145

[B10] Bogden A. E. Carde P. De Paillette E. D. Moreau J. P. Tubiana M. Frindel E. (1991). Amelioration of chemotherapy-induced toxicity by cotreatment with AcSDKP, a tetrapeptide inhibitor of hematopoietic stem cell proliferation. *Ann. N. Y. Acad. Sci.* 628 126–139 10.1111/j.1749-6632.1991.tb17230.x 1648882

[B11] Border W. A. Noble N. A. (1994). Transforming growth factor beta in tissue fibrosis. *N. Engl. J. Med.* 331 1286–1292 10.1056/NEJM199411103311907 7935686

[B12] Brenner B. M. Cooper M. E. De Zeeuw D. Keane W. F. Mitch W. E. Parving H. H. (2001). Effects of losartan on renal and cardiovascular outcomes in patients with type 2 diabetes and nephropathy. *N. Engl. J. Med. * 345 861–869 10.1056/NEJMoa011161 11565518

[B13] Bullard K. M. Longaker M. T. Lorenz H. P. (2003). Fetal wound healing: current biology. *World J. Surg.* 27 54–61 10.1007/s00268-002-6737-2 12557038

[B14] Cashman J. D. Eaves A. C. Eaves C. J. (1994). The tetrapeptide AcSDKP specifically blocks the cycling of primitive normal but not leukemic progenitors in long-term culture: evidence for an indirect mechanism. *Blood* 84 1534–1542 8068944

[B15] Castoldi G. Di Gioia C. R. Bombardi C. Preziuso C. Leopizzi M. Maestroni S. (2013). Renal antifibrotic effect of *N*-acetyl-seryl-aspartyl-lysyl-proline in diabetic rats. *Am. J. Nephrol.* 37 65–73 10.1159/000346116 23327833

[B16] Cavasin M. A. Liao T. D. Yang X. P. Yang J. J. Carretero O. A. (2007). Decreased endogenous levels of Ac-SDKP promote organ fibrosis. *Hypertension* 50 130–136 10.1161/HYPERTENSIONAHA.106.084103 17470726

[B17] Cavasin M. A. Rhaleb N. E. Yang X. P. Carretero O. A. (2004). Prolyl oligopeptidase is involved in release of the antifibrotic peptide Ac-SDKP. *Hypertension* 43 1140–1145 10.1161/01.HYP.0000126172.01673.84 15037553 PMC4677773

[B18] Chen P. L. Chen Y. M. Bookstein R. Lee W. H. (1990). Genetic mechanisms of tumor suppression by the human p53 gene. *Science* 250 1576–1580 10.1126/science.2274789 2274789

[B19] Chen P. Y. Qin L. Barnes C. Charisse K. Yi T. Zhang X. (2012). FGF regulates TGF-beta signaling and endothelial-to-mesenchymal transition via control of let-7 miRNA expression. *Cell Rep.* 2 1684–1696 10.1016/j.celrep.2012.10.021 23200853 PMC3534912

[B20] Chen Y. W. Liu B. W. Zhang Y. J. Chen Y. W. Dong G. F. Ding X. D. (2010). Preservation of basal AcSDKP attenuates carbon tetrachloride-induced fibrosis in the rat liver. *J. Hepatol.* 53 528–536 10.1016/j.jhep.2010.03.027 20646773

[B21] Choi K. Lee K. Ryu S. W. Im M. Kook K. H. Choi C. (2012). Pirfenidone inhibits transforming growth factor-beta1-induced fibrogenesis by blocking nuclear translocation of Smads in human retinal pigment epithelial cell line ARPE-19. *Mol. Vis.* 18 1010–1020 22550395 PMC3339036

[B22] Coates D. Isaac R. E. Cotton J. Siviter R. Williams T. A. Shirras A. (2000). Functional conservation of the active sites of human and *Drosophila* angiotensin I-converting enzyme. *Biochemistry* 39 8963–8969 10.1021/bi000593q 10913309

[B23] Cornell M. J. Williams T. A. Lamango N. S. Coates D. Corvol P. Soubrier F. (1995). Cloning and expression of an evolutionary conserved single-domain angiotensin converting enzyme from *Drosophila melanogaster*. *J. Biol. Chem.* 270 13613–13619 10.1074/jbc.270.23.13613 7775412

[B24] Coutton C. Guigon M. Bohbot A. Ferrani K. Oberling F. (1994). Photoprotection of normal human hematopoietic progenitors by the tetrapeptide N-AcSDKP. *Exp. Hematol.* 22 1076–1080 7925774

[B25] Coward W. R. Saini G. Jenkins G. (2010). The pathogenesis of idiopathic pulmonary fibrosis. *Ther. Adv. Respir. Dis.* 4 367–388 10.1177/1753465810379801 20952439

[B26] Deeg H. J. Seidel K. Hong D. S. Yu C. Huss R. Schuening F. G. (1997). In vivo radioprotective effect of AcSDKP on canine myelopoiesis. *Ann. Hematol.* 74 117–122 10.1007/s002770050268 9111424

[B27] Dooley R. Harvey B. J. Thomas W. (2011). The regulation of cell growth and survival by aldosterone. *Front. Biosci.* 16 440–457 10.2741/3697 21196180

[B28] Ebisawa T. Fukuchi M. Murakami G. Chiba T. Tanaka K. Imamura T. (2001). Smurf1 interacts with transforming growth factor-beta type I receptor through Smad7 and induces receptor degradation. *J. Biol. Chem.* 276 12477–12480 10.1074/jbc.C100008200 11278251

[B29] Esther C. R. Marino E. M. Howard T. E. Machaud A. Corvol P. Capecchi M. R. (1997). The critical role of tissue angiotensin-converting enzyme as revealed by gene targeting in mice. *J. Clin. Invest.* 99 2375–2385 10.1172/JCI119419 9153279 PMC508076

[B30] Feinstein E. Cimino G. Gale R. P. Alimena G. Berthier R. Kishi K. (1991). p53 in chronic myelogenous leukemia in acute phase. *Proc. Natl. Acad. Sci. U.S.A.* 88 6293–6297 10.1073/pnas.88.14.6293 2068108 PMC52069

[B31] Folkman J. (2007). Angiogenesis: an organizing principle for drug discovery? *Nat. Rev. Drug Discov.* 6 273–286 10.1038/nrd2115 17396134

[B32] Fromes Y. Liu J. M. Kovacevic M. Bignon J. Wdzieczak-Bakala J. (2006). The tetrapeptide acetyl-serine-aspartyl-lysine-proline improves skin flap survival and accelerates wound healing. *Wound Repair Regen.* 14 306–312 10.1111/j.1743-6109.2006.00125.x 16808809

[B33] Fuchs S. Frenzel K. Hubert C. Lyng R. Muller L. Michaud A. (2005). Male fertility is dependent on dipeptidase activity of testis ACE. *Nat. Med.* 11 1140–1142 10.1038/nm1105-1140 16270063

[B34] Fuchs S. Xiao H. D. Cole J. M. Adams J. W. Frenzel K. Michaud A. (2004). Role of the N-terminal catalytic domain of angiotensin-converting enzyme investigated by targeted inactivation in mice. *J. Biol. Chem.* 279 15946–15953 10.1074/jbc.M400149200 14757757

[B35] Fuchs S. Xiao H. D. Hubert C. Michaud A. Campbell D. J. Adams J. W. (2008). Angiotensin-converting enzyme C-terminal catalytic domain is the main site of angiotensin I cleavage in vivo. *Hypertension* 51 267–274 10.1161/HYPERTENSIONAHA.107.097865 18158355

[B36] Gieling R. G. Burt A. D. Mann D. A. (2008). Fibrosis and cirrhosis reversibility – molecular mechanisms. *Clin. Liver Dis.* 12 915–937 xi 10.1016/j.cld.2008.07.001 18984474

[B37] Gonzalez G. E. Rhaleb N. E. Nakagawa P. Liao T. D. Liu Y. Leung P. (2014). *N*-acetyl-seryl-aspartyl-lysyl-proline reduces cardiac collagen cross-linking and inflammation in angiotensin II-induced hypertensive rats. *Clin. Sci. (Lond.)* 126 85–94 10.1042/CS20120619 23834332 PMC4240015

[B38] Grande M. T. Lopez-Novoa J. M. (2009). Fibroblast activation and myofibroblast generation in obstructive nephropathy. *Nat. Rev. Nephrol.* 5 319–328 10.1038/nrneph.2009.74 19474827

[B39] Grillon C. Bonnet D. Mary J. Y. Lenfant M. Najman A. Guigon M. (1993). The tetrapeptide AcSerAspLysPro (Seraspenide), a hematopoietic inhibitor, may reduce the in vitro toxicity of 3′-azido-3′-deoxythymidine to human hematopoietic progenitors. *Stem Cells* 11 455–464 10.1002/stem.5530110513 8241956

[B40] Grillon C. Rieger K. Bakala J. Schott D. Morgat J. L. Hannappel E. (1990). Involvement of thymosin beta 4 and endoproteinase Asp-N in the biosynthesis of the tetrapeptide AcSerAspLysPro a regulator of the hematopoietic system. *FEBS Lett.* 274 30–34 10.1016/0014-5793(90)81322-F 2253778

[B41] Gronroos E. Hellman U. Heldin C. H. Ericsson J. (2002). Control of Smad7 stability by competition between acetylation and ubiquitination. *Mol. Cell* 10 483–493 10.1016/S1097-2765(02)00639-1 12408818

[B42] Hannappel E. (2010). Thymosin beta4 and its posttranslational modifications. *Ann. N. Y. Acad. Sci.* 1194 27–35 10.1111/j.1749-6632.2010.05485.x 20536447

[B43] He J. Xu Y. Koya D. Kanasaki K. (2013). Role of the endothelial-to-mesenchymal transition in renal fibrosis of chronic kidney disease. *Clin. Exp. Nephrol.* 17 488–497 10.1007/s10157-013-0781-0 23430391

[B44] Hills C. E. Squires P. E. (2011). The role of TGF-beta and epithelial-to mesenchymal transition in diabetic nephropathy. *Cytokine Growth Factor Rev.* 22 131–139 10.1016/j.cytogfr.2011.06.002 21757394

[B45] Houard X. Williams T. A. Michaud A. Dani P. Isaac R. E. Shirras A. D. (1998). The *Drosophila melanogaster*-related angiotensin-I-converting enzymes Acer and Ance – distinct enzymic characteristics and alternative expression during pupal development. *Eur. J. Biochem.* 257 599–606 10.1046/j.1432-1327.1998.2570599.x 9839949

[B46] Howard T. E. Shai S. Y. Langford K. G. Martin B. M. Bernstein K. E. (1990). Transcription of testicular angiotensin-converting enzyme (ACE) is initiated within the 12th intron of the somatic ACE gene. *Mol. Cell. Biol.* 10 4294–4302 2164636 10.1128/mcb.10.8.4294-4302.1990 PMC360974

[B47] Hubert C. Houot A. M. Corvol P. Soubrier F. (1991). Structure of the angiotensin I-converting enzyme gene. Two alternate promoters correspond to evolutionary steps of a duplicated gene. *J. Biol. Chem.* 266 15377–15383 1651327

[B48] Huff T. Muller C. S. Otto A. M. Netzker R. Hannappel E. (2001). beta-Thymosins, small acidic peptides with multiple functions. *Int. J. Biochem. Cell Biol.* 33 205–220 10.1016/S1357-2725(00)00087-X 11311852

[B49] Ismail-Beigi F. Craven T. Banerji M. A. Basile J. Calles J. Cohen R. M. (2010). Effect of intensive treatment of hyperglycaemia on microvascular outcomes in type 2 diabetes: an analysis of the ACCORD randomised trial. *Lancet* 376 419–430 10.1016/S0140-6736(10)60576-4 20594588 PMC4123233

[B50] Iwamoto N. Xano H. J. Yoshioka T. Shiraga H. Nitta K. Muraki T. (2000). Acetyl-seryl-aspartyl-lysyl-proline is a novel natural cell cycle regulator of renal cells. *Life Sci.* 66 PL221–PL226 10.1016/S0024-3205(00)00460-4 11210724

[B51] Junot C. Gonzales M. F. Ezan E. Cotton J. Vazeux G. Michaud A. (2001). RXP 407, a selective inhibitor of the N-domain of angiotensin I-converting enzyme, blocks in vivo the degradation of hemoregulatory peptide acetyl-Ser-Asp-Lys-Pro with no effect on angiotensin I hydrolysis. *J. Pharmacol. Exp. Ther.* 297 606–611 11303049

[B52] Kanasaki K. Haneda M. Sugimoto T. Shibuya K. Isono M. Isshiki K. (2006). *N*-acetyl-seryl-aspartyl-lysyl-proline inhibits DNA synthesis in human mesangial cells via up-regulation of cell cycle modulators. *Biochem. Biophys. Res. Commun.* 342 758–765 10.1016/j.bbrc.2006.02.019 16497271

[B53] Kanasaki K. Kitada M. Koya D. (2012). Pathophysiology of the aging kidney and therapeutic interventions. *Hypertens. Res.* 35 1121–1128 10.1038/hr.2012.159 23076406

[B54] Kanasaki K. Koya D. Sugimoto T. Isono M. Kashiwagi A. Haneda M. (2003). *N*-Acetyl-seryl-aspartyl-lysyl-proline inhibits TGF-beta-mediated plasminogen activator inhibitor-1 expression via inhibition of Smad pathway in human mesangial cells. *J. Am. Soc. Nephrol.* 14 863–872 10.1097/01.ASN.0000057544.95569.EC 12660320

[B55] Kanasaki K. Taduri G. Koya D. (2013). Diabetic nephropathy: the role of inflammation in fibroblast activation and kidney fibrosis. *Front. Endocrinol. * 4:7 10.3389/fendo.2013.00007 PMC3565176 23390421

[B56] Kanasaki M. Nagai T. Kitada M. Koya D. Kanasaki K. (2011). Elevation of the anti-fibrotic peptide *N*-acetyl-seryl-aspartyl-lysyl-proline: a blood pressure-independent beneficial effect of angiotensin I-converting enzyme inhibitors. *Fibrogenesis Tissue Repair * 4:25 10.1186/1755-1536-4-25 PMC3253677 22126210

[B57] Koutrafouri V. Leondiadis L. Avgoustakis K. Livaniou E. Czarnecki J. Ithakissios D. S. (2001). Effect of thymosin peptides on the chick chorioallantoic membrane angiogenesis model. *Biochim. Biophys. Acta* 1568 60–66 10.1016/S0304-4165(01)00200-8 11731086

[B58] Krege J. H. John S. W. Langenbach L. L. Hodgin J. B. Hagaman J. R. Bachman E. S. (1995). Male-female differences in fertility and blood pressure in ACE-deficient mice. *Nature* 375 146–148 10.1038/375146a0 7753170

[B59] Kroger W. L. Douglas R. G. O’Neill H. G. Dive V. Sturrock E. D. (2009). Investigating the domain specificity of phosphinic inhibitors RXPA380 and RXP407 in angiotensin-converting enzyme. *Biochemistry* 48 8405–8412 10.1021/bi9011226 19658433

[B60] Kurisaki A. Kose S. Yoneda Y. Heldin C. H. Moustakas A. (2001). Transforming growth factor-beta induces nuclear import of Smad3 in an importin-beta1 and Ran-dependent manner. *Mol. Biol. Cell* 12 1079–1091 10.1091/mbc.12.4.1079 11294908 PMC32288

[B61] Kwakernaak A. J. Waanders F. Slagman M. C. Dokter M. M. Laverman G. D. De Boer R. A. (2013). Sodium restriction on top of renin–angiotensin-aldosterone system blockade increases circulating levels of *N*-acetyl-seryl-aspartyl-lysyl-proline in chronic kidney disease patients. *J. Hypertens.* 31 2425–2432 10.1097/HJH.0b013e328364f5de 24029871

[B62] Lan H. Y. (2011). Diverse roles of TGF-beta/Smads in renal fibrosis and inflammation. *Int. J. Biol. Sci.* 7 1056–1067 10.7150/ijbs.7.1056 21927575 PMC3174390

[B63] Langford K. G. Shai S. Y. Howard T. E. Kovac M. J. Overbeek P. A. Bernstein K. E. (1991). Transgenic mice demonstrate a testis-specific promoter for angiotensin-converting enzyme. *J. Biol. Chem.* 266 15559–15562 1651914

[B64] LeBleu V. S. Taduri G. O’Connell J. Teng Y. Cooke V. G. Woda C. (2013). Origin and function of myofibroblasts in kidney fibrosis. *Nat. Med.* 19 1047–1053 10.1038/nm.3218 23817022 PMC4067127

[B65] Lenfant M. Wdzieczak-Bakala J. Guittet E. Prome J. C. Sotty D. Frindel E. (1989). Inhibitor of hematopoietic pluripotent stem cell proliferation: purification and determination of its structure. *Proc. Natl. Acad. Sci. U.S.A.* 86 779–782 10.1073/pnas.86.3.779 2915977 PMC286560

[B66] Lewis E. J. Hunsicker L. G. Bain R. P. Rohde R. D. (1993). The effect of angiotensin-converting-enzyme inhibition on diabetic nephropathy. The Collaborative Study Group. * N. Engl. J. Med. * 329:1456–1462 10.1056/NEJM199311113292004 8413456

[B67] Li P. Xiao H. D. Xu J. Ong F. S. Kwon M. Roman J. (2010). Angiotensin-converting enzyme N-terminal inactivation alleviates bleomycin-induced lung injury. *Am. J. Pathol.* 177 1113–1121 10.2353/ajpath.2010.081127 20651228 PMC2928946

[B68] Lin C. X. Rhaleb N. E. Yang X. P. Liao T. D. D’Ambrosio M. A. Carretero O. A. (2008). Prevention of aortic fibrosis by *N*-acetyl-seryl-aspartyl-lysyl-proline in angiotensin II-induced hypertension. *Am. J. Physiol. Heart Circ. Physiol.* 295 H1253–H1261 10.1152/ajpheart.00481.2008 18641275 PMC2544498

[B69] Liu J. M. Bignon J. Ilic V. Briscoe C. Lallemand J. Y. Riches A. (2006). Evidence for an association of high levels of endogenous Acetyl-Ser-Asp-Lys-Pro, a potent mediator of angiogenesis, with acute myeloid leukemia development. *Leuk. Lymphoma* 47 1915–1920 10.1080/10428190600688131 17065006

[B70] Liu J. M. Garcia-Alvarez M. C. Bignon J. Kusinski M. Kuzdak K. Riches A. (2010). Overexpression of the natural tetrapeptide acetyl-*N*-ser-asp-lys-pro derived from thymosin beta4 in neoplastic diseases. *Ann. N. Y. Acad. Sci.* 1194 53–59 10.1111/j.1749-6632.2010.05488.x 20536450

[B71] Liu J. M. Gora-Tybor J. Grzybowska-Izydorczyk O. Bignon J. Robak T. Wdzieczak-Bakala J. (2009a). Elevated plasma levels of the angiogenic tetrapeptide acetyl-ser-asp-lys-pro are found in some patients with hematologic malignancies. *Leuk. Lymphoma* 50 2096–2097 10.3109/10428190903331074 19863169

[B72] Liu Y. H. D’Ambrosio M. Liao T. D. Peng H. Rhaleb N. E. Sharma U. (2009b). *N*-acetyl-seryl-aspartyl-lysyl-proline prevents cardiac remodeling and dysfunction induced by galectin-3, a mammalian adhesion/growth-regulatory lectin. *Am. J. Physiol. Heart Circ. Physiol.* 296 H404–H412 10.1152/ajpheart.00747.2008 19098114 PMC2643891

[B73] Liu J. M. Kusinski M. Ilic V. Bignon J. Hajem N. Komorowski J. (2008). Overexpression of the angiogenic tetrapeptide AcSDKP in human malignant tumors. *Anticancer. Res.* 28 2813–2817 19035315

[B74] Liu J. M. Lawrence F. Kovacevic M. Bignon J. Papadimitriou E. Lallemand J. Y. (2003). The tetrapeptide AcSDKP, an inhibitor of primitive hematopoietic cell proliferation, induces angiogenesis in vitro and in vivo. *Blood* 101 3014–3020 10.1182/blood-2002-07-2315 12480715

[B75] Liu Y. (2011). Cellular and molecular mechanisms of renal fibrosis. *Nat. Rev. Nephrol.* 7 684–696 10.1038/nrneph.2011.149 22009250 PMC4520424

[B76] Macconi D. Tomasoni S. Romagnani P. Trionfini P. Sangalli F. Mazzinghi B. (2012). MicroRNA-324-3p promotes renal fibrosis and is a target of ACE inhibition. *J. Am. Soc. Nephrol.* 23 1496–1505 10.1681/ASN.2011121144 22822076 PMC3431411

[B77] Mackensen-Haen S. Bader R. Grund K. E. Bohle A. (1981). Correlations between renal cortical interstitial fibrosis, atrophy of the proximal tubules and impairment of the glomerular filtration rate. *Clin. Nephrol.* 15 167–171 7237863

[B78] Macours N. Hens K. (2004). Zinc-metalloproteases in insects: ACE and ECE. *Insect Biochem. Mol. Biol.* 34 501–510 10.1016/j.ibmb.2004.03.007 15147752

[B79] Malinda K. M. Goldstein A. L. Kleinman H. K. (1997). Thymosin beta 4 stimulates directional migration of human umbilical vein endothelial cells. *FASEB J.* 11 474–481 9194528 10.1096/fasebj.11.6.9194528

[B80] Meran S. Steadman R. (2011). Fibroblasts and myofibroblasts in renal fibrosis. *Int. J. Exp. Pathol.* 92 158–167 10.1111/j.1365-2613.2011.00764.x 21355940 PMC3101489

[B81] Miyazono K. (2000). TGF-beta signaling by Smad proteins. *Cytokine Growth Factor Rev.* 11 15–22 10.1016/S1359-6101(99)00025-8 10708949

[B82] Nagai T. Kanasaki M. Srivastava S. Nakamura Y. Ishigaki Y. Kitada M. (2014). *N*-acetyl-seryl-aspartyl-lysyl-proline inhibits diabetes-associated kidney fibrosis and endothelial–mesenchymal transition. *Biomed. Res. Int.* 2014:696475 10.1155/2014/696475 PMC3982268 24783220

[B83] Nakagawa P. Liu Y. Liao T. D. Chen X. Gonzalez G. E. Bobbitt K. R. (2012). Treatment with *N*-acetyl-seryl-aspartyl-lysyl-proline prevents experimental autoimmune myocarditis in rats. *Am. J. Physiol. Heart Circ. Physiol.* 303 H1114–H1127 10.1152/ajpheart.00300.2011 22923621 PMC3517643

[B84] Nakao A. Afrakhte M. Moren A. Nakayama T. Christian J. L. Heuchel R. (1997). Identification of Smad7, a TGFbeta-inducible antagonist of TGF-beta signalling. *Nature* 389 631–635 10.1038/39369 9335507

[B85] Nath K. A. (1992). Tubulointerstitial changes as a major determinant in the progression of renal damage. *Am. J. Kidney Dis.* 20 1–17 1621674 10.1016/s0272-6386(12)80312-x

[B86] Nyberg P. Xie L. Kalluri R. (2005). Endogenous inhibitors of angiogenesis. *Cancer Res.* 65 3967–3979 10.1158/0008-5472.CAN-04-2427 15899784

[B87] Ohkubo Y. Kishikawa H. Araki E. Miyata T. Isami S. Motoyoshi S. (1995). Intensive insulin therapy prevents the progression of diabetic microvascular complications in Japanese patients with non-insulin-dependent diabetes mellitus: a randomized prospective 6-year study. *Diabetes. Res. Clin. Pract.* 28 103–117 10.1016/0168-8227(95)01064-K 7587918

[B88] Omata M. Taniguchi H. Koya D. Kanasaki K. Sho R. Kato Y. (2006). *N*-acetyl-seryl-aspartyl-lysyl-proline ameliorates the progression of renal dysfunction and fibrosis in WKY rats with established anti-glomerular basement membrane nephritis. *J. Am. Soc. Nephrol.* 17 674–685 10.1681/ASN.2005040385 16452498

[B89] Parving H. H. (2001). Diabetic nephropathy: prevention and treatment. *Kidney Int.* 60 2041–2055 10.1046/j.1523-1755.2001.00020.x 11703631

[B90] Peng H. Carretero O. A. Liao T. D. Peterson E. L. Rhaleb N. E. (2007). Role of *N*-acetyl-seryl-aspartyl-lysyl-proline in the antifibrotic and anti-inflammatory effects of the angiotensin-converting enzyme inhibitor captopril in hypertension. *Hypertension* 49 695–703 10.1161/01.HYP.0000258406.66954.4f 17283252 PMC3257515

[B91] Peng H. Carretero O. A. Peterson E. L. Rhaleb N. E. (2010). Ac-SDKP inhibits transforming growth factor-beta1-induced differentiation of human cardiac fibroblasts into myofibroblasts. *Am. J. Physiol. Heart Circ. Physiol.* 298 H1357–H1364 10.1152/ajpheart.00464.2009 20154264 PMC2867434

[B92] Peng H. Carretero O. A. Raij L. Yang F. Kapke A. Rhaleb N. E. (2001). Antifibrotic effects of *N*-acetyl-seryl-aspartyl-Lysyl-proline on the heart and kidney in aldosterone-salt hypertensive rats. *Hypertension* 37 794–800 10.1161/01.HYP.37.2.794 11230375 PMC6824419

[B93] Philp D. Huff T. Gho Y. S. Hannappel E. Kleinman H. K. (2003). The actin binding site on thymosin beta4 promotes angiogenesis. *FASEB J.* 17 2103–2105 10.1096/fj.03-0121fje 14500546

[B94] Pokharel S. Rasoul S. Roks A. J. Van Leeuwen R. E. Van Luyn M. J. Deelman L. E. (2002). *N*-acetyl-Ser-Asp-Lys-Pro inhibits phosphorylation of Smad2 in cardiac fibroblasts. *Hypertension* 40 155–161 10.1161/01.HYP.0000025880.56816.FA 12154106

[B95] Pradelles P. Frobert Y. Creminon C. Ivonine H. Frindel E. (1991). Distribution of a negative regulator of haematopoietic stem cell proliferation (AcSDKP) and thymosin beta 4 in mouse tissues. *FEBS Lett.* 289 171–175 10.1016/0014-5793(91)81062-D 1915845

[B96] Pradelles P. Frobert Y. Creminon C. Liozon E. Masse A. Frindel E. (1990). Negative regulator of pluripotent hematopoietic stem cell proliferation in human white blood cells and plasma as analysed by enzyme immunoassay. *Biochem. Biophys. Res. Commun.* 170 986–993 10.1016/0006-291X(90)90489-A 2202303

[B97] RamachandraRao S. P. Zhu Y. Ravasi T. Mcgowan T. A. Toh I. Dunn S. R. (2009). Pirfenidone is renoprotective in diabetic kidney disease. *J. Am. Soc. Nephrol.* 20 1765–1775 10.1681/ASN.2008090931 19578007 PMC2723978

[B98] Redd M. J. Cooper L. Wood W. Stramer B. Martin P. (2004). Wound healing and inflammation: embryos reveal the way to perfect repair. *Philos. Trans. R. Soc. Lond. B Biol. Sci.* 359 777–784 10.1098/rstb.2004.1466 15293805 PMC1693361

[B99] Remuzzi G. Schieppati A. Ruggenenti P. (2002). Clinical practice. Nephropathy in patients with type 2 diabetes. *N. Engl. J. Med.* 346 1145–1151 10.1056/NEJMcp011773 11948275

[B100] Rhaleb N. E. Peng H. Harding P. Tayeh M. Lapointe M. C. Carretero O. A. (2001a). Effect of *N*-acetyl-seryl-aspartyl-lysyl-proline on DNA and collagen synthesis in rat cardiac fibroblasts. *Hypertension* 37 827–832 10.1161/01.HYP.37.3.827 11244003 PMC6824426

[B101] Rhaleb N. E. Peng H. Yang X. P. Liu Y. H. Mehta D. Ezan E. (2001b). Long-term effect of *N*-acetyl-seryl-aspartyl-lysyl-proline on left ventricular collagen deposition in rats with 2-kidney, 1-clip hypertension. *Circulation* 103 3136–3141 10.1161/01.CIR.103.25.3136 11425781 PMC4679287

[B102] Risdon R. A. Sloper J. C De Wardener H. E. (1968). Relationship between renal function and histological changes found in renal biopsy specimens from patients with persistent glomerular nephritis. *Lancet * 2:363–366 10.1016/S0140-6736(68)90589-8 4173786

[B103] Ritz E. Rychlik I. Locatelli F. Halimi S. (1999). End-stage renal failure in type 2 diabetes: a medical catastrophe of worldwide dimensions. *Am. J. Kidney Dis.* 34 795–808 10.1016/S0272-6386(99)70035-1 10561134

[B104] Rodriguez-Iturbe B Garcia Garcia G. (2010). The role of tubulointerstitial inflammation in the progression of chronic renal failure. *Nephron Clin. Pract.* 116 c81–c88 10.1159/000314656 20502043

[B105] Rousseau A. Michaud A. Chauvet M. T. Lenfant M. Corvol P. (1995). The hemoregulatory peptide *N*-acetyl-Ser-Asp-Lys-Pro is a natural and specific substrate of the N-terminal active site of human angiotensin-converting enzyme. *J. Biol. Chem.* 270 3656–3661 10.1074/jbc.270.8.3656 7876104

[B106] Schainuck L. I. Striker G. E. Cutler R. E. Benditt E. P. (1970). Structural–functional correlations in renal disease. II. The correlations. *Hum. Pathol.* 1 631–641 10.1016/S0046-8177(70)80061-2 5521736

[B107] Sharma K. Ix J. H. Mathew A. V. Cho M. Pflueger A. Dunn S. R. (2011). Pirfenidone for diabetic nephropathy. *J. Am. Soc. Nephrol.* 22 1144–1151 10.1681/ASN.2010101049 21511828 PMC3103734

[B108] Sharma U. Rhaleb N. E. Pokharel S. Harding P. Rasoul S. Peng H. (2008). Novel anti-inflammatory mechanisms of *N*-Acetyl-Ser-Asp-Lys-Pro in hypertension-induced target organ damage. *Am. J. Physiol. Heart Circ. Physiol.* 294 H1226–H1232 10.1152/ajpheart.00305.2007 18178715 PMC6824420

[B109] Shibuya K. Kanasaki K. Isono M. Sato H. Omata M. Sugimoto T. (2005). *N*-acetyl-seryl-aspartyl-lysyl-proline prevents renal insufficiency and mesangial matrix expansion in diabetic db/db mice. *Diabetes Metab. Res. Rev.* 54 838–845 10.2337/diabetes.54.3.838 15734863

[B110] Smart N. Risebro C. A. Melville A. A. Moses K. Schwartz R. J. Chien K. R. (2007). Thymosin beta4 induces adult epicardial progenitor mobilization and neovascularization. *Nature* 445 177–182 10.1038/nature05383 17108969

[B111] Srivastava S. P. Koya D. Kanasaki K. (2013). MicroRNAs in kidney fibrosis and diabetic nephropathy: roles on EMT and EndMT. *Biomed. Res. Int.* 2013:125469 10.1155/2013/125469 PMC3780472 24089659

[B112] Stephan J. Melaine N. Ezan E. Hakovirta H. Maddocks S. Toppari J. (2000). Source, catabolism and role of the tetrapeptide *N*-acetyl-ser-asp-lys-Pro within the testis. *J. Cell Sci. * 113(Pt 1) 113–121 10591630 10.1242/jcs.113.1.113

[B113] Striker G. E. Schainuck L. I. Cutler R. E. Benditt E. P. (1970). Structural–functional correlations in renal disease. I. A method for assaying and classifying histopathologic changes in renal disease. *Hum. Pathol.* 1 615–630 10.1016/S0046-8177(70)80060-0 5521735

[B114] Strutz F. Zeisberg M. (2006). Renal fibroblasts and myofibroblasts in chronic kidney disease. *J. Am. Soc. Nephrol.* 17 2992–2998 10.1681/ASN.2006050420 17035610

[B115] Suzuki C. Murakami G. Fukuchi M. Shimanuki T. Shikauchi Y. Imamura T. (2002). Smurf1 regulates the inhibitory activity of Smad7 by targeting Smad7 to the plasma membrane. *J. Biol. Chem.* 277 39919–39925 10.1074/jbc.M201901200 12151385

[B116] Takakuta K. Fujimori A. Chikanishi T. Tanokura A. Iwatsuki Y. Yamamoto M. (2010). Renoprotective properties of pirfenidone in subtotally nephrectomized rats. *Eur. J. Pharmacol.* 629 118–124 10.1016/j.ejphar.2009.12.011 20006961

[B117] The Diabetes Control and Complications Trial Research Group. (1993). The effect of intensive treatment of diabetes on the development and progression of long-term complications in insulin-dependent diabetes mellitus. *N. Engl. J. Med.* 329 977–986 10.1056/NEJM199309303291401 8366922

[B118] UK Prospective Diabetes Study (UKPDS) Group. (1998). Effect of intensive blood-glucose control with metformin on complications in overweight patients with type 2 diabetes (UKPDS 34). *Lancet* 352 854–865 10.1016/S0140-6736(98)07037-8 9742977

[B119] Vazeux G. Cotton J. Cuniasse P. Dive V. (2001). Potency and selectivity of RXP407 on human, rat, and mouse angiotensin-converting enzyme. *Biochem. Pharmacol.* 61 835–841 10.1016/S0006-2952(01)00550-0 11274969

[B120] Vegter S. Perna A. Postma M. J. Navis G. Remuzzi G. Ruggenenti P. (2012). Sodium intake, ACE inhibition, and progression to ESRD. *J. Am. Soc. Nephrol.* 23 165–173 10.1681/ASN.2011040430 22135311 PMC3269916

[B121] Viswanathan V. (1999). Type 2 diabetes and diabetic nephropathy in India – magnitude of the problem. *Nephrol. Dial. Transplant.* 14 2805–2807 10.1093/ndt/14.12.2805 10570073

[B122] Waeckel L. Bignon J. Liu J. M. Markovits D. Ebrahimian T. G. Vilar J. (2006). Tetrapeptide AcSDKP induces postischemic neovascularization through monocyte chemoattractant protein-1 signaling. *Arterioscler. Thromb. Vasc. Biol.* 26 773–779 10.1161/01.ATV.0000203510.96492.14 16410461

[B123] Wang D. Carretero O. A. Yang X. Y. Rhaleb N. E. Liu Y. H. Liao T. D. (2004). *N*-acetyl-seryl-aspartyl-lysyl-proline stimulates angiogenesis in vitro and in vivo. *Am. J. Physiol. Heart Circ. Physiol.* 287 H2099–H2105 10.1152/ajpheart.00592.2004 15256375 PMC6824423

[B124] Wang M. Liu R. Jia X. Mu S. Xie R. (2010). *N*-acetyl-seryl-aspartyl-lysyl-proline attenuates renal inflammation and tubulointerstitial fibrosis in rats. *Int. J. Mol. Med.* 26 795–801 21042772 10.3892/ijmm_00000527

[B125] Watanabe T. Brown G. S. Kelsey L. S. Yan Y. Jackson J. D. Ewel C. (1996). In vivo protective effects of tetrapeptide AcSDKP, with or without granulocyte colony-stimulation factor, on murine progenitor cells after sublethal irradiation. *Exp. Hematol.* 24 713–721 8635527

[B126] Wdzieczak-Bakala J. Fache M. P. Lenfant M. Frindel E. Sainteny F. (1990). AcSDKP, an inhibitor of CFU-S proliferation, is synthesized in mice under steady-state conditions and secreted by bone marrow in long-term culture. *Leukemia* 4 235–237 2314120

[B127] Wei L. Alhenc-Gelas F. Corvol P. Clauser E. (1991). The two homologous domains of human angiotensin I-converting enzyme are both catalytically active. *J. Biol. Chem.* 266 9002–9008 1851160

[B128] Wierenga P. K. Brenner M. K. Konings A. W. (1998). Enhanced selectivity of hyperthermic purging of human progenitor cells using Goralatide, an inhibitor of cell cycle progression. *Bone Marrow Transplant.* 21 73–78 10.1038/sj.bmt.1701045 9486498

[B129] Wierenga P. K. Konings A. W. (1994). Seraspenide (AcSDKP) mediated protection of hematopoietic stem cells in a hyperthermic purging protocol. *Prog. Clin. Biol. Res.* 389 189–195 7700902

[B130] Wierenga P. K. Setroikromo R. Vellenga E. Kampinga H. H. (2000). Purging of acute myeloid leukaemia cells from stem cell grafts by hyperthermia: enhancement of the therapeutic index by the tetrapeptide AcSDKP and the alkyl-lysophospholipid ET-18-OCH(3). *Br. J. Haematol.* 111 1145–1152 10.1046/j.1365-2141.2000.02469.x 11167754

[B131] Wrana J. L. Attisano L. Carcamo J. Zentella A. Doody J. Laiho M. (1992). TGF beta signals through a heteromeric protein kinase receptor complex. *Cell* 71 1003–1014 10.1016/0092-8674(92)90395-S 1333888

[B132] Wynn T. A. (2007). Common and unique mechanisms regulate fibrosis in various fibroproliferative diseases. *J. Clin. Invest.* 117 524–529 10.1172/JCI31487 17332879 PMC1804380

[B133] Xiao Z. Liu X. Lodish H. F. (2000). Importin beta mediates nuclear translocation of Smad 3. *J. Biol. Chem.* 275 23425–23428 10.1074/jbc.C000345200 10846168

[B134] Xu H. Yang F. Sun Y. Yuan Y. Cheng H. Wei Z. (2012). A new antifibrotic target of Ac-SDKP: inhibition of myofibroblast differentiation in rat lung with silicosis. *PLoS ONE * 7:e40301 10.1371/journal.pone.0040301 PMC3389005 22802960

[B135] Yang F. Yang X. P. Liu Y. H. Xu J. Cingolani O. Rhaleb N. E. (2004). Ac-SDKP reverses inflammation and fibrosis in rats with heart failure after myocardial infarction. *Hypertension* 43 229–236 10.1161/01.HYP.0000107777.91185.89 14691195 PMC3259854

[B136] Zeisberg M. Duffield J. S. (2010). Resolved: EMT produces fibroblasts in the kidney. *J. Am. Soc. Nephrol.* 21 1247–1253 10.1681/ASN.2010060616 20651165

[B137] Zisman L. S. (1998). Inhibiting tissue angiotensin-converting enzyme: a pound of flesh without the blood? *Circulation* 98 2788–2790 10.1161/01.CIR.98.25.2788 9860776

[B138] Zuo Y. Chun B. Potthoff S. A. Kazi N. Brolin T. J. Orhan D. (2013). Thymosin beta4 and its degradation product, Ac-SDKP, are novel reparative factors in renal fibrosis. *Kidney Int.* 84 1166–1175 10.1038/ki.2013.209 23739235 PMC3830708

